# The CA3 region of the hippocampus: how is it? What is it for? How does it do it?

**DOI:** 10.3389/fncel.2015.00019

**Published:** 2015-02-05

**Authors:** Enrico Cherubini, Richard Miles

**Affiliations:** ^1^Department of Neuroscience, International School for Advanced StudiesTrieste, Italy; ^2^European Brain Research Institute (EBRI) Rita Levi-MontalciniRome, Italy; ^3^Institut du Cerveau et de la Moelle, CHU Pitié-Salpêtrière Inserm U1127, CNRS UMR7225, Université Pierre et Marie Curie UMR S1127Paris, France

**Keywords:** hippocampus, CA3 subfield, mossy fibers, associative network, theta rhythm, spatial representation, episodic memory, Aβ peptide

The hippocampus, in the temporal lobe, is phylogenetically one of the oldest parts of the brain and forms part of the limbic system. The hippocampus proper is defined by the dentate gyrus and Cornu Ammonis (CA). While the dentate gyrus contains the fascia dentata and the hilus, the CA is anatomically and functionally differentiated into distinct subfields named CA1, CA2, CA3, and CA4. The CA3 region has attracted major attention in recent years for its specific role in memory processes, susceptibility to seizures and neuro-degeneration.

Internal connectivity in the CA3 subfield is more rich than in other hippocampal regions. Recurrent axon collaterals of CA3 pyramidal cells ramify extensively making excitatory contacts with neighboring excitatory and inhibitory neurons (Lorente de Nó, 1934). This circuit is implicated in encoding spatial representations (O'Keefe and Nadel, 1978) and episodic memories (Scoville and Milner, 1957). It generates coherent population synchronies, including gamma, theta and sharp-waves, presumed to associate firing in selected assemblies of cells in different behavioral conditions (Buzsáki et al., 1983). The CA3 region receives inputs from the entorhinal cortex either directly *via* the perforant path or indirectly from the dentate gyrus *via* the mossy fibers (Amaral and Witter, 1983). Mossy fiber connections made with principal or mossy cells terminate with large boutons (5–8 μm) while those with interneurons are targeted by smaller filopodial extensions. The mossy fiber pathway acts as a high-pass filter that translates densely coded cortical signals to a sparse, specific hippocampal code, essential for memory formation.

This e-book aims to highlight recent advances by bringing together experts on the cellular and molecular mechanisms regulating the wiring properties of the CA3 hippocampal microcircuit in both physiological and pathological conditions. The seven reviews and four research articles are organized to follow neuronal information flowing from the dentate gyrus to the CA3 associative network.

Firstly, Münster-Wandowski et al. (2013) (*Inst. Integrative Neuroanatomy, La Charité, Berlin and Instituto Politécnico Nacional, Mexico City*) review recent data showing that MF terminals can transiently release GABA as well as glutamate, both in early post-natal life and after periods of intense activity in adulthood. GABA and glutamate co-release from single identified MF boutons has been recently demonstrated. Mossy fibers apparently form electrical synapses, as well as chemical synapses, with CA3 pyramidal cells potentially permitting a fast excitation to overcome a strong, but delayed, feed-forward inhibition.

Since MF axons of dentate granule cells may be generated post-natally, Pedroni et al. (2014) (*Dept. Neurosci, SISSA, Trieste and European Brain Research Inst, Rome*) studied immature granule cells at P0–P3. At this age, with a clear GABAergic phenotype, granule cells have small somata, few dendritic branches and axons often terminate as growth cones in the CA3 region. Depolarization induces either rudimentary or overshooting sodium spikes and low threshold calcium spikes. Post-synaptic CA3 pyramidal cells participate in spontaneous GDPs driven by synergistic actions of GABA and glutamate. The excitatory actions of GABA released from mossy fiber terminals may be crucial for network synchrony, suggested to refine microcircuits of the CA3 region.

Radial glial cells guide migrating CA3 neurons during development. After characterizing signaling molecules and the temporal sequence of hippocampal development, Belvindrah et al. (2014) (*INSERM U839, Inst. Fer à Moulin, Paris*) review abnormalities of migration linked to human brain malformations: agenesia, lissencephaly, holoprosencephaly, polymicrogyria, heterotopia, and focal cortical dysplasia. Mutations in genes associated with neuronal migration in mouse models result in lamination defects of the CA3 area, a region especially vulnerable to stress and seizure-induced damage. This review links genetic, migration and cellular defects and multiple inherited neurological and psychiatric disorders.

An overview of how CA3 neurons process afferent activity from dentate granule cells is provided by Evstratova and Tóth (2014) (*Dept. Psychiatry and Neuroscience, Université Laval, Quebec City*). They describe how the shape of mossy fiber terminals affects transmission to both CA3 pyramidal cells and interneurons. Transmission exhibits short-term plasticities over different frequencies which vary according to post-synaptic cell type. Granule cells usually fire at 0.01–0.1 Hz, but frequency can increase to 15–50 Hz or even higher when spatial information is coded. At these higher frequencies, glutamate also activates presynaptic autoreceptors, which modulate calcium signaling and release from mossy fiber terminals. These processes, including synaptic plasticity, differ at MF.

Lorente de Nó (1934) suggested that recurrent cortical connections might underly reverberating neuronal discharges as a short-term electrical memory. Le Duigou et al. (2014) (*INSERM U112, Inst. du Cerveau et de la Moelle, Paris*) review recurrent circuits in the CA3 region. CA3 pyramidal cell axons form an associative network associated with sharp waves and other EEG oscillations as well as epileptiform synchrony. Paired records from CA3 pyramidal cells and interneurons have provided data on synaptic contacts and efficacy within recurrent circuits. Connectivity in the associative recurrent CA3 network seems to be spatially more extensive and sparse than in other sensory cortices possibly facilitating representation coding.

Kesner (2013) (*Dept. Psychology, Univ. Utah, Salt Lake City*) reviews contributions from the CA3 region subfields CA3a, b and c in acquiring and encoding spatial information. Mnemonic functions depend on synaptic interactions of CA3 associative networks, operating as an attractor, with inputs from the dentate gyrus and entorhinal cortex (Kesner and Hunsaker, 2010). Fields CA3a and b encode spatial information in short-term memory and also support retrieval by spatial pattern completion. In contrast, the CA3c field may support pattern separation *via* interactions with the DG. The output subfields, CA3a and b process information sequentially communicating with the CA1 region *via* the Schaffer collaterals.

Rolls (2013) (*Centre for Computational Neuroscience, Univ Oxford and Dept. Computer Science, Univ. Warwick*) describes quantitatively how the CA3 region operates and how it contributes to episodic memory. The CA3 system, through its recurrent collateral connections, is presented as a single attractor enabling fast, one trial, associations between any spatial location and an object or reward. A recurrent structure permits memory completion during recall from any subset of acquired links. This theory permits associations between time, objects and rewards to provide the temporal order needed for episodic memory. Neurophysiological tests and supports of the theory are described with novel hypotheses on the advantages of a low recurrent connectivity in CA3.

Cerasti and Treves (2013) (*Cognitive Neuroscience, SISSA, Trieste, Collège de France, Paris and Kavli Inst., Trondheim*) next discuss how self-organizing recurrent connections enable spatial representations to be acquired in the CA3 area. A simplified network model shows that self-organization can lead to the emergence of multiple loosely organized discrete point-like attractors which differ from structures associated with a single, continuous attractor.

The pivotal role of the CA3 region in humans is discussed by Deuker et al. (2014) (*Dept. Epileptology and Center for Neurodegenerative Diseases Univ. Bonn and Donders Inst., Univ. Nijmegen*). They describe how the human CA3 region forms associations during encoding and how during retrieval it reconstructs memory representations with pattern separation and completion based on partial cues (Deuker et al., 2013). Functional properties of different hippocampal subfields can be identified with high resolution fMRI and related to behavioral, and structural alterations associated with mild cognitive impairment in early Alzheimer's disease.

Spatial information is encoded by the phase of principal cell firing during theta oscillations. The hyperpolarization activated cation current I_h_ is a critical regulator of this phase dependent firing. The research article of Borel et al. (2013) (*Dept. Physiology, Development and Neuroscience, Univ. Cambridge and Dept. Brain and Cognitive Engineering, Korea Univ. Seoul*) asks how I_h_ controls firing of CA1 and CA3 pyramidal cells. CA1 neurons are suggested to express I_h_ at higher levels than CA3 pyramidal cells ensuring larger responses to hyperpolarization and thus more prominent resonance peaks. With a strong I_h_, excitatory inputs during theta can delay firing, an effect counteracted by phasic inhibitory currents during theta oscillations. Interactions between synaptic inputs and active I_h_ currents may then account for distinct phase responses of CA1 and CA3 neurons in temporal outputs during theta oscillations.

The final article from Nava-Mesa et al. (2013) (Lab. Neurofisiología y Comportamiento, Univ. Castilla-La Mancha, Ciudad Real and Dept. Fisiología y Farmacología, Univ. Salamanca) shows how the Aβ peptide affects synapses made by fimbrial afferents with CA3 pyramidal cells. Stimulation of the lateral fimbria evokes AMPA-mediated EPSPs followed by early and late IPSPs mediated by distinct GABA receptors. Aβ depolarizes CA3 pyramidal cells, increases their input resistance and decreases the late GABA_B_-mediated IPSP current. This post-synaptic effect, is mediated via the G protein-gated inwardly rectifying potassium channel GirK. Dysfunction of septo-hippocampal oscillations involving GirK at fimbria-CA3 synapses may account for memory deficits during early stages of Alzheimer's disease.

## Conflict of interest statement

The authors declare that the research was conducted in the absence of any commercial or financial relationships that could be construed as a potential conflict of interest.
